# In Situ Photodegradation of Incorporated Polyanion Does Not Alter Prion Infectivity

**DOI:** 10.1371/journal.ppat.1002001

**Published:** 2011-02-03

**Authors:** Justin R. Piro, Brent T. Harris, Surachai Supattapone

**Affiliations:** 1 Department of Biochemistry, Dartmouth Medical School, Hanover, New Hampshire, United States of America; 2 Department of Pathology, Dartmouth Medical School, Hanover, New Hampshire, United States of America; 3 Department of Medicine, Dartmouth Medical School, Hanover, New Hampshire, United States of America; Istituto Superiore di Sanità, Italy

## Abstract

Single-stranded polyanions ≥40 bases in length facilitate the formation of hamster scrapie prions *in vitro*, and polyanions co-localize with PrP^Sc^ aggregates *in vivo* [1], [2]. To test the hypothesis that intact polyanionic molecules might serve as a structural backbone essential for maintaining the infectious conformation(s) of PrP^Sc^, we produced synthetic prions using a photocleavable, 100-base oligonucleotide (PC-oligo). In serial Protein Misfolding Cyclic Amplification (sPMCA) reactions using purified PrP^C^ substrate, PC-oligo was incorporated into physical complexes with PrP^Sc^ molecules that were resistant to benzonase digestion. Exposure of these nuclease-resistant prion complexes to long wave ultraviolet light (315 nm) induced degradation of PC-oligo into 5 base fragments. Light-induced photolysis of incorporated PC-oligo did not alter the infectivity of *in vitro*-generated prions, as determined by bioassay in hamsters and brain homogenate sPMCA assays. Neuropathological analysis also revealed no significant differences in the neurotropism of prions containing intact versus degraded PC-oligo. These results show that polyanions >5 bases in length are not required for maintaining the infectious properties of *in vitro*-generated scrapie prions, and indicate that such properties are maintained either by short polyanion remnants, other co-purified cofactors, or by PrP^Sc^ molecules alone.

## Author Summary

Prions are unorthodox infectious agents whose composition remains undetermined. Previous experiments have shown that long, negatively charged polymers such as nucleic acid and carbohydrate molecules promote the formation of purified prions in test tube chemical reactions. Various classes of negatively charged polymers have also been found to co-exist within prion complexes in the brains of infected animals. These observations suggest that negatively charged polymers might act as a structural support necessary for prion infectivity. We tested this possibility by chemically synthesizing a negatively charged polymer that can be degraded by exposure to ultraviolet light. Prions containing this light-sensitive polymer remained infectious after light exposure, indicating that negatively charged polymers are not necessary to maintain the structural shapes of infectious prions.

## Introduction

Infectious prion diseases such as Creutzfeldt Jakob disease (CJD) and other related human disorders, chronic wasting disease (CWD), bovine spongiform encephalopathy (BSE), and scrapie are associated with the conversion of a host-encoded glycoprotein (PrP^C^) into a misfolded conformer, PrP^Sc^ [3]. Several biochemical studies utilizing the protein misfolding cyclic amplification (PMCA) technique have shown that PrP^Sc^ is an essential component of the infectious agent [4], [5], [6].

Interestingly, a variety of prion strains with distinctive infectious phenotypes, such as selective neurotropism and characteristic disease incubation times, have been isolated and propagated *in vivo* and *in vitro* [7], [8]. In some cases, infection with specific prion strains produces PrP^Sc^ molecules with distinctive biochemical characteristics, suggesting that multiple self-propagating PrP^Sc^ conformations may provide the structural basis for the existence of multiple prion strains [9], [10].

Several biochemical and cell culture studies have implicated polyanions such as single stranded nucleic acid and glycosaminoglycan (GAG) molecules as potent cofactors in the process of infectious prion propagation [1], [5], [6], [11], [12], [13], [14], [15], [16], [17]. Moreover, it has been shown that polyanions are selectively incorporated into physical complexes with purified PrP molecules in infectious hamster prions, and that a minimum length corresponding to a 40 base single stranded oligonucleotide is required for a polyanion to facilitate hamster prion formation *in vitro* [1]. Neuropathological analysis of scrapie-infected animals has shown that nucleic acids and GAG-containing proteoglycans co-localize with PrP^Sc^ aggregates *in situ* [1], [2]. Collectively, these observations raise the possibility that moderately long polyanions might be needed to maintain hamster prion infectivity or strain properties by acting as a structural support for PrP^Sc^ molecules.

Nuclease-resistant RNA molecules have been detected in purified prion preparations [18], and analytical methods suggest that the average size of polynucleotides in such preparations is ≤25 nucleotides, assuming a ratio of 1 polynucleotide molecule per infectious unit [19]. More recently, Jeong *et al.* reported reduction of prion infectivity and PrP^Sc^ by treatment of hamster 263K scrapie brain homogenates with LiAlH_4_ (lithium aluminum hydride), a highly reactive reducing agent capable of degrading RNA molecules [20]. However, because LiAlH_4_ is a non-specific reducing agent, which can react with a variety of biological molecules other than RNA, including proteins, it is not possible to ascribe definitively the detrimental effect of LiAlH_4_ on prion infectivity to the degradation of RNA.

Previous photo-irradiation studies of naturally occurring prions [21], [22] suggested that specific nucleic acid sequences are not necessary for prion infectivity, and this conclusion was confirmed by the *de novo* formation of purified prions using synthetic, homopolymeric poly(A) RNA molecules [5]. However, these prior studies did not exclude a structural, non-coding role for polyanions such as RNA in maintaining the infectious conformation of PrP^Sc^ since ultraviolet (UV) light mutates pyrimidine bases but does not significantly degrade the polynucleotide backbone of nucleic acids or other types of polyanions such as GAGs.

Here we report the results of studies using purified synthetic prions containing a photocleavable (dT)_100_ oligonucleotide (PC-oligo), which can be selectively degraded *in situ* into 5-mers by exposure to long wave UV light. This approach directly addresses the question of whether polyanions might serve as a structural backbone for infectious prions in a chemically defined model system.

## Materials and Methods

### Reagents

Hamster prion strain Sc237 was kindly provided by Dr. Stanley Prusiner (UCSF, San Francisco, CA). Three-week-old female Golden Syrian hamsters used in inoculation experiments and seven-week-old Golden Syrian hamsters used to make brain homogenates were purchased from Charles River (Wilmington, MA). Monoclonal antibody (mAb) 6D11 was purchased from Covance (Princeton, NJ). PC-oligo was chemically synthesized by placing a photocleavable group in between every five base of (dT)100, using (3-(4,4′-Dimethoxytrityl)-1-(2-nitrophenyl)-propan-1-yl-[(2-cyanoethyl)-(N,N-diisopropyl)]-phosphoramidite). PC-oligowas synthesized by and purchased from Gene Link (Hawthorne, NY). Stock solutions of PC-oligo were made by dissolving lyophilized powder into 50% DMSO/ 50% TE pH 8.0 (10 mM Tris 8.0, 1 mM EDTA) to a final concentration of 5 mg/ml. The dT-oligo was synthesized by and purchased from Operon (Huntsville, AL). Wild type mouse recombinant PrP was expressed in *E. Coli* and purified as previously described [6]. Benzonase nuclease (50-230-8706) was purchased from EMD Chemicals (Gibbstown, NH).

### Serial Protein Misfolding Cyclic Amplification (sPMCA)

sPMCA reactions were performed as previously described [4], [5]. Reaction tubes were subjected to PMCA for 24 h using a Misonix s3000 programmable sonicator equipped with a microplate horn (Misonix, Farmingdale, NY) containing 350 ml of water. The temperature was maintained inside the horn by a circulating water bath, pumping warmed water through aluminum coils surrounding the horn. Sample tubes were held in a plastic rack which prevents lid opening and hold tubes ∼3 mm from the surface of the horn. After 24 h, the tubes were removed from the sonicator, and 10 µl of the reaction mixture was added to a new tube containing fresh substrate.

### Western Blotting

A portion of each sample was treated with 25 µg/ml proteinase K for 30 min at 37°C shaking at 750 rpm in an Eppendorf Thermomixer (Fisher Scientific, Pittsburg, PA). An equal volume of 2x sodium dodecyl sulfate (SDS) loading buffer was added to each tube and then boiled at 95°C for 10 min. SDS-polyacrylamide gel electrophoresis (PAGE) was then performed using 1.5 mm 12% polyacrylamide gels (29∶1 acrylamide∶bisacrylamide) The gel was transferred to a methanol-charged polyvinylidene difluoride membrane (Millipore, Billerica, MA) using a Transblot SD semidry transfer cell (Bio-Rad, Hercules, CA). The transfer was set to achieve 3 mA/cm^2^ for 30 min. After transfer, the membrane was incubated in 20% (v/v) instant nonfat dry milk (Nestle, Vevey, Switzerland) dissolved in TBST (10 mM Tris pH 7.1, 150 mM NaCl, 0.1% Tween 20). The membrane was then incubated overnight at 4°C with mAb 6D11 in TBST (final concentration 80 ng/ml). The membrane was washed 3 times with TBST for 10 min and then incubated for 1 h with horseradish peroxidase-labeled anti-mouse immunoglobulin G secondary antibody conjugate (GE Healthcare) diluted 1∶5,000 in TBST. Again the membrane was washed 4 times for 10 min in TBST. Blots were developed with West Pico (Pierce, Rockford, IL) chemiluminescence substrate. Images were captured using a Fuji LAS-3000 chemiluminescence documentation system (Fujifilm, Tokyo, Japan). Relative molecular masses were determined by comparison to prestained standards from Fermentas (Hanover, MD).

### Preparation of Purified PrP^Sc^ Molecules

Immunopurified PrP^C^ was prepared as described previously [5]. To produce substrate mixtures, 37.5 µl of immunopurified PrP^C^ was mixed with 10 µl of 10x reaction buffer (200 mM MOPS pH 7.5, 10% Triton X-100, 500 mM imidazole, 50 mM EDTA, 1.5 M NaCl), 7.5 µl of dT-oligo or PC-oligo (0.3 mg/ml for a total of 2.25 µg) and 35 µl of molecular grade water (Mediatech, Herndon, VA). On the first day, 10 µl of Sc237 brain homogenate was added to a 0.5 ml thin-walled PCR tube (Axygen, Union City, CA) and subjected to sPMCA as described above. The sonicator delivered bursts of 30 s every 30 min at output 6.5. sPMCA was continued for 15 days, effectively diluting the original inocula 10^15^ fold. The negative control line was treated as stated above with the omission of nucleic acid from the substrate cocktail.

### Light Treatment of Oligonucleotides

dT-oligo and PC-oligo were placed in their own ProxiPlate 96-well plate (PerkinElmer, Waltham, MA). The plates were placed into a UV Stratalinker 2400 (Stratagene, La Jolla, CA) equipped with bulbs that emit light at 315 nm. The 96-well plates were placed on top of tip boxes and racks so samples were ∼2.5 cm from the light source. Each light treated sample received 4 pulses of light at an energy level of 500,000 µJ. Non-light treated samples were also placed in their own ProxiPlate 96-well plate but were then placed inside a drawer to protect from light. Treated and non-treated oligos were either added to sPMCA reactions as described above, or directly analyzed as described below. For benzonase protection assays 6 µg of either dT-oligo or PC-oligo were incubated for 10 min at 37°C and shaking at 750 rpm with or without 300 µg recPrP. After incubation samples were sonicated for one 30 s pulse followed by another 10 min incubation at 37°C and 750 rpm. After second incubation 7 µl of benzonase was added (175 units) and further Incubated for 1 h at 37°C and 750 rpm. The oligonucleotides were then extracted as described below.

### Light Treatment of PrP^Sc^ Samples

Concentrations of PrP^Sc^ containing either dT-oligo or PC-oligo were compared by Western blot and diluted to achieve equal concentrations. Of the normalized material, 500 µl of each sample was placed in its own ProxiPlate 96-well plate (PerkinElmer, Waltham, MA). Samples were then treated as described above with one modification. In between each light pulse, samples were removed from the 96-well plate, placed in a 1.5 ml tube and subjected to sonication in a cold sonicator. Briefly, 350 ml of chilled water was placed in the microplate horn, and tubes were positioned in a rack as described above and subjected to a 20 s pulse at output 6.5. Samples were returned to new wells in the 96-well plate and subjected to another round of light or dark treatment.

### DNA Recovery and Analysis

To analyze the effect of light on the nucleic acids 150 µl of each sample was brought to 250 µl by adding 1x reaction buffer. An equal volume of Phenol∶Chloroform∶Isoamyl alcohol 25∶24∶1 (Sigma, St. Louis, MO) was added to each tube and vortexed. Samples were spun at 14,000× *g* for 2 min. The top aqueous phase was carefully removed and the process repeated twice more. After three extractions with the Phenol∶Chloroform∶Isoamyl alcohol, an equal volume of chloroform was added to each tube, vortexed and spun as above. The top aqueous phase was removed and to it 25 µl of 3 M sodium acetate pH 7.0 was added followed by 750 µl of 95% EtOH. Tubes were vortexed and incubated at −20°C for 15 min. After incubation tubes were spun at 14,000× *g* for 10 min to pellet the DNA. The pellet was washed with 80% EtOH, spun and dried. The pellet was resuspended in equal volumes of TE (10 mM Tris pH 7.0, 1 mM EDTA) and 2x TBE-Urea sample buffers (Invitrogen, Carlsbad, CA). Samples were boiled at 95°C for 5 min and then loaded into pre-cast 15% TBE-Urea acrylamide gels (Invitrogen, Carlsbad, CA). Gels were run in a X-Cell *Sure*Lock apparatus (Invitrogen, Carlsbad, CA) at 200 V for 50 min. Molecular weight ladder was made by mixing oligo dT 100mer, oligo dT 30mer and oligo dT 10mer (10 mg/ml) in a ratio of 1∶3∶9 with 2x TBE-Urea sample buffer (oligos purchased from Operon, Huntsville, AL). Gels were then stained for 1 h with Sybr Gold (Invitrogen, Carlsbad, CA) diluted 1∶10,000 in TBE (89 mM Tris-base, 89 mM Boric Acid, 2 mM EDTA pH 8.3). After staining, gels were visualized on a transilluminator and photographed with a Canon Power Shot A650 IS (Canon, Tokyo, Japan) equipped with proper filters.

### sPMCA End Point Titration

A 10% hamster brain homogenate (w/v) was made in conversion buffer (PBS with 150 mM NaCl, 1% Triton X-100, 4 mM EDTA) plus a Complete PI tablet with EDTA (Poche, Indianapolis, IN) using a polytron tissue grinder. The homogenate was then spun at 200× *g* for 30 s. The supernatant was then aliquotted into 0.5 ml thin-walled PCR tubes (90 µl reactions) and frozen at −70°C until used. PrP^Sc^ molecules treated with or without light were serially diluted into conversion buffer (PBS with 150 mM NaCl, 1% Triton X-100, 5 mM EDTA) to create 10 titers ranging from 10^−1^–10^−10^. Each titer was used to start a reaction by adding 10 µl to 90 µl of brain homogenate prepared as described above. Samples were subjected to 3 rounds of sPMCA as described above. The sonicator for this experiment was set to deliver 30 s pulses at output 8.5. Each sample was Western blotted as described above.

### Preparation of Inocula

For each of the lines injected, 20 µl of day 15 material was spiked into each of 5 reaction tubes containing 200 µl of fresh substrate cocktail and subjected to 1 cycle of PMCA as described above. After 24 h tubes were removed and combined. This material was then treated with or without light as described above. After treatments, 100 µl of each sample was diluted into 900 µl of sterile PBS with 5 mg/ml bovine serum albumin (BSA) to prepare the inoculum. The DNA alone inoculum was prepared by mixing 2.25 µg of each nucleic acid into 100 µl of 1x reaction buffer. This material was then diluted 1∶10 into 5 mg/ml BSA, as described above.

### Scrapie Inoculation and Diagnosis

Injections were performed in laminar-flow biosafety cabinets using disposable syringes, gloves and surfaces. Intracerebral inoculations were performed using 28-gauge hypodermic needles inserted into the parietal lobe. Each animal received 50 µl of inoculum. Hamsters were examined daily by veterinary staff blinded to experimental groups. Standard diagnostic criteria were used to identify animals showing signs of scrapie.

### Neuropathology

Animals were euthanized by CO_2_ inhalation until death was imminent. Their brains were quickly removed using sterile dissection instruments on disposable surfaces. Brains were immersed in 10% buffered formalin for 1 week. Sagittal and parasagittal sections were made and brains placed into tissue-processing cassettes. The cassettes were immersed into 90% formic acid for 1 hr and then placed into 10% formalin. Tissues were next embedded in paraffin and cut into 4 µm sections. Sections were stained with hematoxylin and eosin. A single neuropathologist, who was blinded to the experimental groups examined all slides and scored them according to the degree of vacuolation. Scores were made according to previously published standards [5].

### Ethics Statement

All animals were handled in strict accordance with good animal practice, as defined by the Guide for the Care and Use of Laboratory Animals of the National Institutes of Health. The Dartmouth College Institutional Animal Care and Use Committee approved the animal work (assurance number A3259-01). Inoculations were performed under isoflurane anesthesia, and all efforts were made to minimize suffering.

## Results

**Figure 1:**
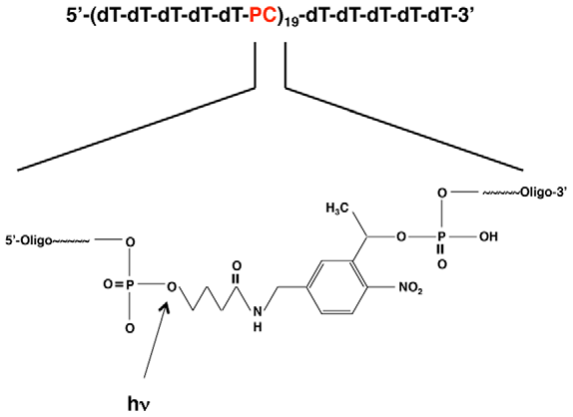
Schematic showing the composition of the PC-oligo. Repeating units of five deoxythymine residues were followed by a photocleavable (PC) linker as shown. Enlarged inset shows the chemical structure of the PC linker (3-(4,4′-Dimethoxytrityl)-1-(2-nitrophenyl)-propan-1-yl-[(2-cyanoethyl)-(N,N-diisopropyl)]-phosphoramidite). The arrow indicates the location of the cleavage site (the phosphodiester bond between the linker and the 5′ phosphate of the oligonucleotide).

We chemically synthesized a 100-base poly-dT molecule containing with a UV-photocleavable group (PC-oligo) every five bases (Figure 1). To confirm that PC-oligo can support PrP^Sc^ propagation, sPMCA reactions containing purified Syrian hamster PrP^C^ supplemented with either PC-oligo or dT-oligo were seeded with Sc237 brain homogenate. The PrP^C^ substrate used in these studies has been previously characterized extensively by a variety of analytical methods, and was found to contain stoichiometric quantities of co-purified endogenous lipids; but no other proteins, nucleic acids, or specific metal ions [5]. The results of our preliminary experiment show that both PC-oligo and dT-oligo support PrP^Sc^ propagation through three rounds of sPMCA reactions whereas control reactions without added polyanions failed to propagate (Figure S1 in Supporting Information S1). After proteinase K (PK) digestion, PrP^Sc^ molecules formed with either oligonucleotide showed a similar shift in electrophoretic mobility to 27–30 kD (Figure S1 in Supporting Information S1).

To determine the optimal irradiation conditions for the photolysis of PC-oligo, pure solutions of each oligonucleotide in buffer were treated with varying amounts of UV light (315 nm) and analyzed by acrylamide gel electrophoresis. Prior to light treatment, PC-oligo can be seen a 5-base ladder, representing the collection of full length and incomplete products formed during the sequential conjugation chemical synthesis protocol (Figure S2 in Supporting Information S1, lane 3). Upon exposure to two Joules of light, PC-oligo was completely degraded to 5-base oligonucleotide units (Figure S2 in Supporting Information S1, compare lanes 3 and 7) while dT-oligo remained intact (Figure S2 in Supporting Information S1, compare lanes 1 and 2).

**Figure 2:**
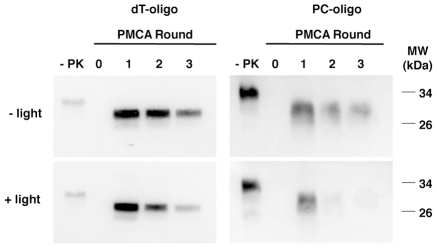
Western blot showing the effect of degrading PC-oligo on its ability to stimulate prion conversion in sPMCA. Lanes 1 and 6 show non-digested PrP^C^ used as substrate (-PK). The remaining samples were subjected to limited proteolysis with proteinase K.

We next tested the effect of light-induced degradation on the ability of PC-oligo to facilitate prion propagation in sPMCA reactions. As anticipated, dT-oligo supported propagation of Sc237 prions in sPMCA reactions using immunopurified hamster PrP^C^ substrate even when the control oligonucleotide was pretreated with UV light (Figure 2, left gels). PC-oligo pretreated with UV light did not support propagation (Figure 2, bottom right gel), whereas PC-oligo mock-treated in the dark supported Sc237 propagation in sPMCA for three rounds (Figure 2, top right gel). These results confirm our previously published results that an oligonucleotide must be at least 40 bases long to support propagation, and that our light treatment protocol completely degrades PC-oligo to a length smaller than that required for efficient propagation in sPMCA.

**Figure 3:**
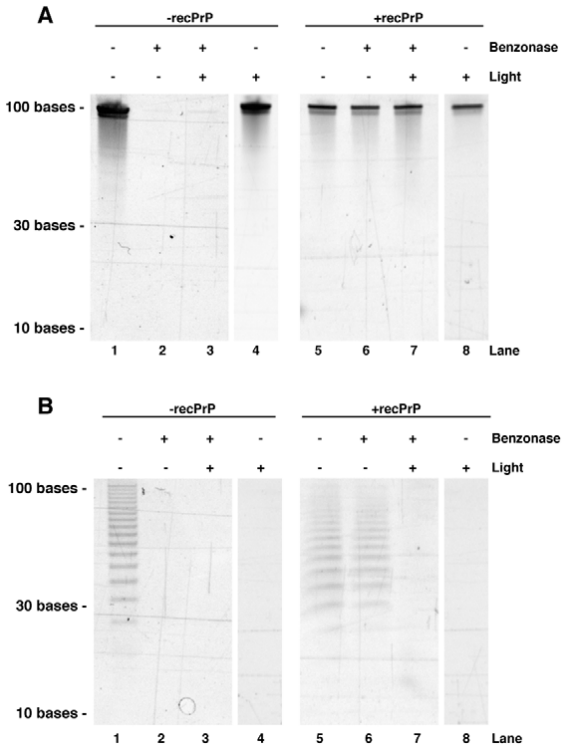
Acrylamide gel electrophoresis showing the effects of benzonase and light treatment on oligonucleotide integrity. **A.** Experiments performed with dT-oligo. **B.** Experiments performed with PC-oligo. In both panels, treatments were performed on oligonucleotides in the presence or absence of 1.5 mg/ml recPrP, as indicated.

We previously found that single stranded nucleic acids form a nuclease-resistant complex with both PrP^C^ and PrP^Sc^ molecules during sPMCA reactions[1]. To study the penetration of UV light into nucleoprotein complexes, we combined a stoichiometric excess of full-length recombinant mouse PrP (recPrP) with our test oligonucleotides. Following PMCA sonication, both PC-oligo and dT-oligo became completely protected from nuclease digestion, whereas both oligonucleotides were nuclease-sensitive in the absence of PrP (Figures 3A and 3B). UV irradiation induced the degradation of PC-oligo, but not dT-oligo, within these nuclease-resistant complexes containing excess PrP (compare Figure 3A, lanes 7–8 and Figure 3B, lanes 7–8). Thus, UV light is able to penetrate PrP nucleoprotein complexes, providing us with a unique opportunity to control the degradation of PC-oligo *in situ* within purified prions.

**Figure 4:**
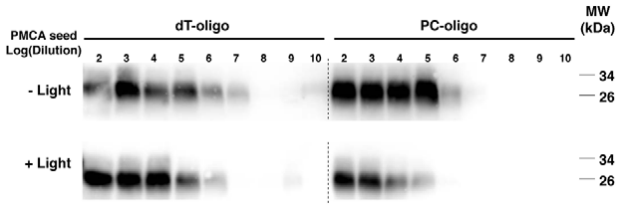
Western blot showing the final samples from 3 round sPMCA reactions. Samples containing dT-oligo or PC-oligo were seeded with a dilution of PrP^Sc^ which was either subjected to light treatment or not, as indicated. All samples were subjected to limited proteolysis with proteinase K.

Having established conditions for UV light penetration of PrP nucleoprotein complexes, we sought to determine the effect of light-induced degradation of PC-oligo *in situ* (i.e. already incorporated into complexes PrP^Sc^ molecules) on the conformational stability, sPMCA seeding activity, and biological infectivity of synthetic PrPSc molecules. PrP^Sc^ molecules containing either PC-oligo or control dT-oligo were generated by performing sPMCA for 15 rounds in order to dilute the initial prion seeds several orders of magnitude beyond the calculated endpoint [4], [5], and then either treated with UV light or mock-treated in the dark. A PK digestion assay revealed no significant differences in PrP^Sc^ protease-resistance between light-treated versus mock-treated samples (Figure S3 in Supporting Information S1). To measure sPMCA seeding activity, samples were serially titrated, and the resulting dilutions were used to seed three rounds of sPMCA using fresh hamster brain homogenate as substrate, and samples from the final round of sPMCA were analyzed for the presence of PrP^Sc^ by Western blot. The results of this quantitative end-point titration assay show that PrP^Sc^ molecules containing either dT-oligo or PC-oligo were equally susceptible to UV irradiation; both sets of samples displayed ∼1 log decrease in seed titer upon light treatment, presumably due to non-specific effects (Figure 4). A small amount of each sample was concurrently analyzed by acrylamide gel electrophoresis to confirm that UV treatment was successful in degrading the PC-oligo below detection limits (Figure S4 in Supporting Information S1). Taken together, the results indicate that light-induced degradation of incorporated PC-oligo into 5-mers had no specific effect on the ability of PrP^Sc^ to seed sPMCA reactions of normal brain homogenate.

**Figure 5:**
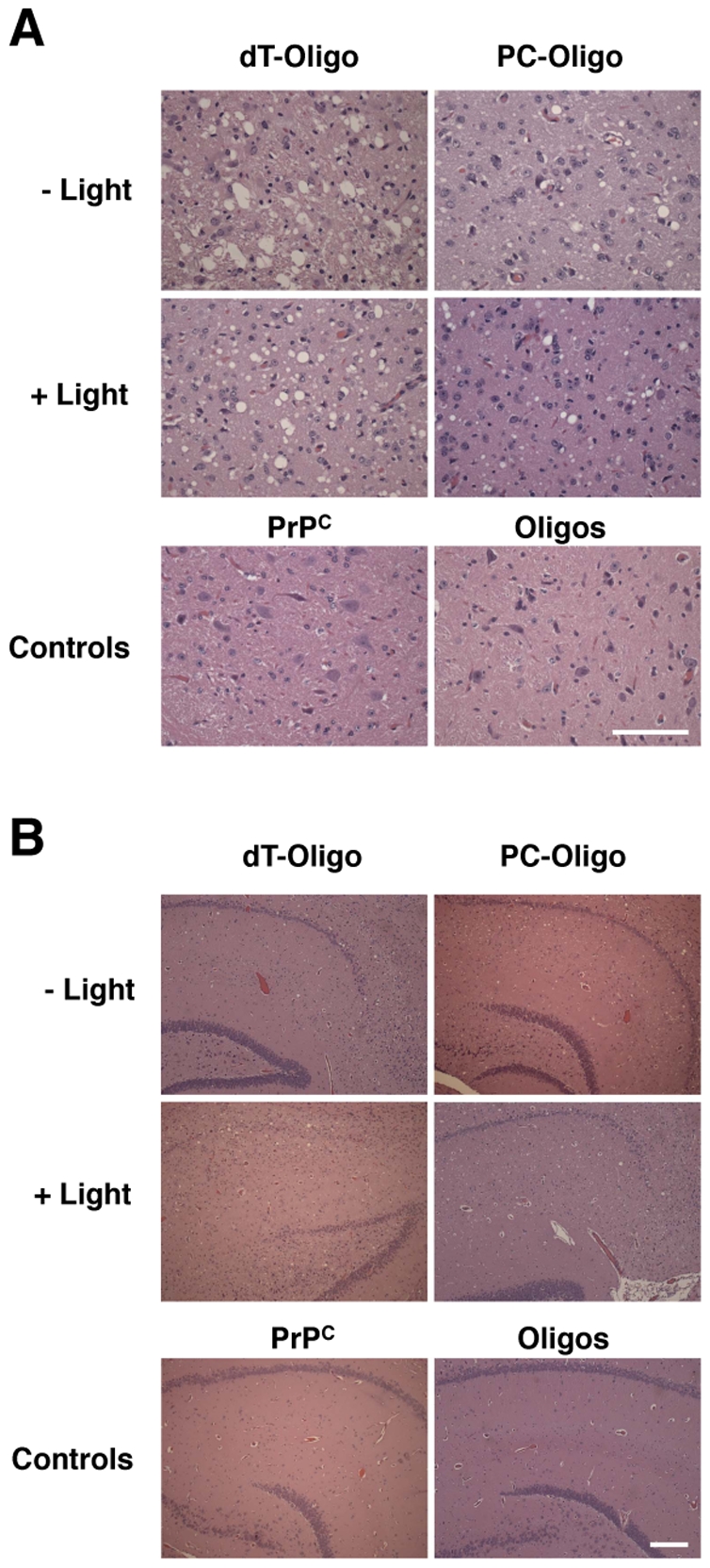
Representative histological fields of brainstem and hippocampus. Hemotoxylin and Eosin staining from animals injected with *in vitro* generated PrP^Sc^ containing dT-oligo or PC-oligo treated with or without light. Top row: animals injected with prions treated in dark. Middle row: animals injected with prions treated with light. Bottom row: control animals. **A.** Brain stem. (Scale bar, 100 µm). **B.** Hippocampus. (Scale bar, 200 µm).

**Figure 6:**
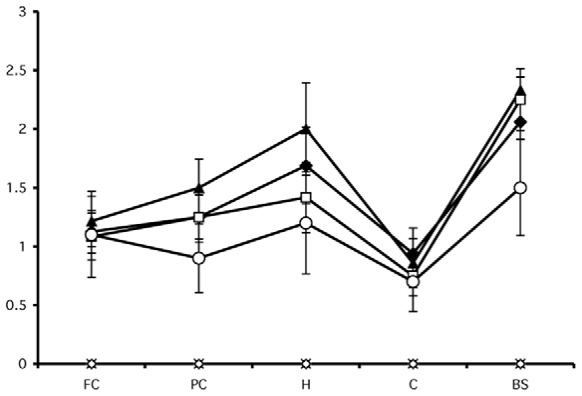
Regional neuropathology of hamsters infected with light- or dark-treated inocula. Vacuolation profile scores of animals inoculated with samples containing dark-treated dT-oligo (-◆-), light-treated dT-oligo (-⌑-), dark-treated PC-oligo (-▲-), light-treated PC-oligo (-◯-), PrP^C^ (-X-), or dT/PC-oligos alone (-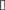-). The mean values (n = 5–8 animals/group) are shown ± S.E.M. FC: Frontal cortex. PC: Parietal cortex. H: Hippocampus. C: Cerebellum. BS: Brain stem.

Light- and dark-treated PrP^Sc^ containing either dT-oligo or PC-oligo were injected intracerebrally into Golden Syrian hamsters for bioassay. In addition, negative control samples consisting of either the original Sc237 seed propagated for 15 rounds in PrP^C^ substrate without nucleic acid (designated PrP^C^) or a cocktail containing the dT-oligo and PC-oligo (designated dT/PC-oligo alone) were inoculated in parallel. The scrapie incubation time for all 4 experimental groups was ∼150 days, with all of the inoculated animals developing similar clinical signs of ataxia, trembling, and circling (Table 1). Animals injected with the PrP^C^ and dT/PC-oligo control samples did not develop signs of clinical scrapie during the time frame of the experiment (Table 1 and figures 5A and 5B). Neuropathological analysis showed significant vacuolization throughout the brains of animals in all 4 groups, including the brainstem and hippocampus (Figure 5A and 5B), and a blinded comparison of vacuolization scores from different brain regions showed no difference between the experimental groups (Figure 6). Biochemical analysis showed no differences in the protease sensitivity, glycoform ratio, or electrophoretic mobility of the PrP^Sc^ molecules produced in the brains of animals from all 4 experimental groups (Figure S5 in Supporting Information S1). There was also no apparent difference in the conformational stability of PrP^Sc^ molecules in the brains of animals injected with light-treated versus control inocula, as determined by a urea denaturation assay (Figure S6 in Supporting Information S1). Therefore, these results indicate that degradation of an incorporated polyanion does not significantly alter the biological infectivity or the strain-dependent properties of purified Sc237 prions.

**Table 1:** Prion incubation times in hamsters inoculated intracerebrally with sPMCA generated prions.

Inoculum	n/n_0_	Incubation Time (Days ± S.E.M.)
dT-Oligo–light	8/8	153±1
dT-Oligo+light	6/6	153±0
PC-Oligo–light	7/7	151±1
PC-Oligo+light	5/5	153±0
PrP^C^	0/4	>182
dT/PC-Oligo alone	0/4	>182

The PrP^C^ sample was generated by serially propagating the original Sc237 seed for 15 rounds in PrP^C^ substrate alone (without polyanion) at a 1∶10 ratio in each round. The dT/PC-Oligo alone inoculum contains a mixture of both oligonucleotides as described in Methods.

## Discussion

The major finding of this work is that selective degradation of an incorporated photocleavable polyanion cofactor does not alter the catalytic activity, infectivity, or strain properties of *in vitro* generated prions.

These results contrast with those of Jeong *et al.*, who showed that treatment of scrapie-infected brain homogenates with LiAlH_4_ caused degradation of endogenous RNA and increased prion incubation time from ∼100 to ∼340 days [20]. There are several possible reasons for the discrepancy between the two studies: (1) Although both LiAlH_4_ and UV light treatment are capable of reacting non-specifically with non-polyanionic molecules, under the conditions used for each study, it is possible that LiAlH_4_ treatment is more non-specifically damaging than UV light. For instance, it could reduce PrP^Sc^ molecules and/or directly damage protein structure. (2) The work of Jeong *et al.* used scrapie brain homogenate as the starting infectious material, whereas the experiments reported here used purified prions formed from native PrP^C^ substrate *in vitro* using sPMCA. Such purified prions are composed only of PrP, stoichiometric amounts of an endogenous lipid molecule containing 20 carbon fatty acids, and a synthetic polyanion [5]. (3) Whereas LiAlH_4_ is capable of hydrolyzing RNA molecules completely, UV light degrades the PC-oligo used in our study to (dT)_5_ oligonucleotides. Although such small nucleic acids do not support prion propagation *in vitro*, our results cannot formally exclude the possibility that short oligonucleotides, once incorporated, might be able to act as a reinforcing backbone for PrP^Sc^. (4) Although we did not detect any intact PC-oligo after light treatment using a highly sensitive intercalating agent, it is possible that a small, undetectable amount of PC-oligo was protected from UV-irradiation by virtue of being complexed with PrP^Sc^ molecules. This possibility is difficult to address experimentally because it is technically difficult to recover and detect such small amounts of DNA that cannot be amplified. It is worth noting that UV light degrades PC-oligo quantitatively within nuclease-resistant complexes with recombinant PrP.

Our results are compatible with the scenario in which prion infectivity might be exclusively encoded by PrP^Sc^ structure in the absence of non-proteinaceous cofactors, as proposed by the “protein only” hypothesis [23], [24]. However, this hypothesis cannot be confirmed in our experimental system because both endogenous lipid and remnant (dT)_5_ oligonucleotide molecules remain present in purified prions generated with PC-oligo following light treatment. Nonetheless, we are able to conclude that it is unnecessary for prions to contain polyanions >5 bases in length to maintain infectivity, whereas polyanions ≥40 bases are required for the process of prion formation *in vitro*. This conclusion places a significant geometric constraint on the mechanism by which polyanionic cofactors could be theoretically used to maintain PrP^Sc^ architecture. Stoichiometric quantities of endogenous lipids containing 20-carbon fatty acids co-purify with the PrP^C^ substrate used in these studies [5], and therefore these lipid molecules could also become incorporated into the infectious PrP^Sc^ product during PMCA reactions. Future studies will be required to test whether endogenous lipid molecules or other non-nucleic acid cofactors, such as those recently described in mouse brain homogenates [25], [26], might be required to maintain prion infectivity and strain phenotypes.

## Supporting Information

Supporting Information S1 Supporting Figures S1 through S6 Found at: doi:10.1371/journal.ppat.1002001.s001
(2.3MB DOC)
