# Supplementary material for: In Situ Photodegradation of Incorporated Polyanion Does Not Alter Prion Infectivity
Source: PLoS Pathog. 2011 Feb 3;7(2):e1002001. doi: 10.1371/journal.ppat.1002001 (PMC3033378; doi:10.1371/journal.ppat.1002001)
Supplement: Supporting Information S1 — Supporting Figures S1 through S6 Found at: doi:10.1371/journal.ppat.1002001.s001 (2.3MB DOC) [file ppat.1002001.s001.doc]

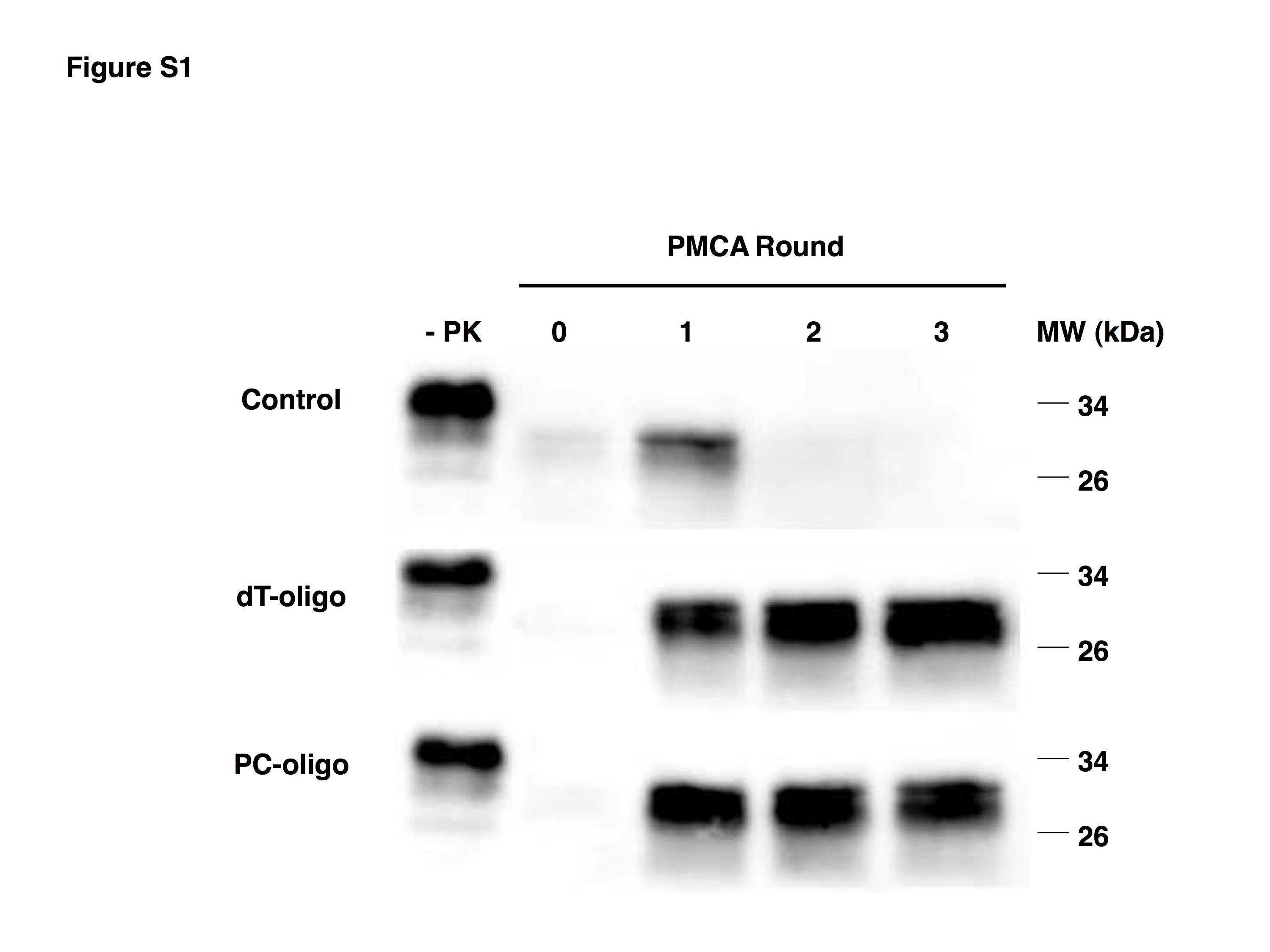


**Figure S1.** Western blot showing sPMCA reactions using immunopurified HaPrP^C^ as substrate. Reactions were supplemented with 22.5 μg/ml oligonucleotide or water (control), as indicated. Lane 1 shows non-digested PrP^C^ used as a substrate in sPMCA reactions (-PK). The remaining samples were subjected to limited proteolysis with proteinase K.


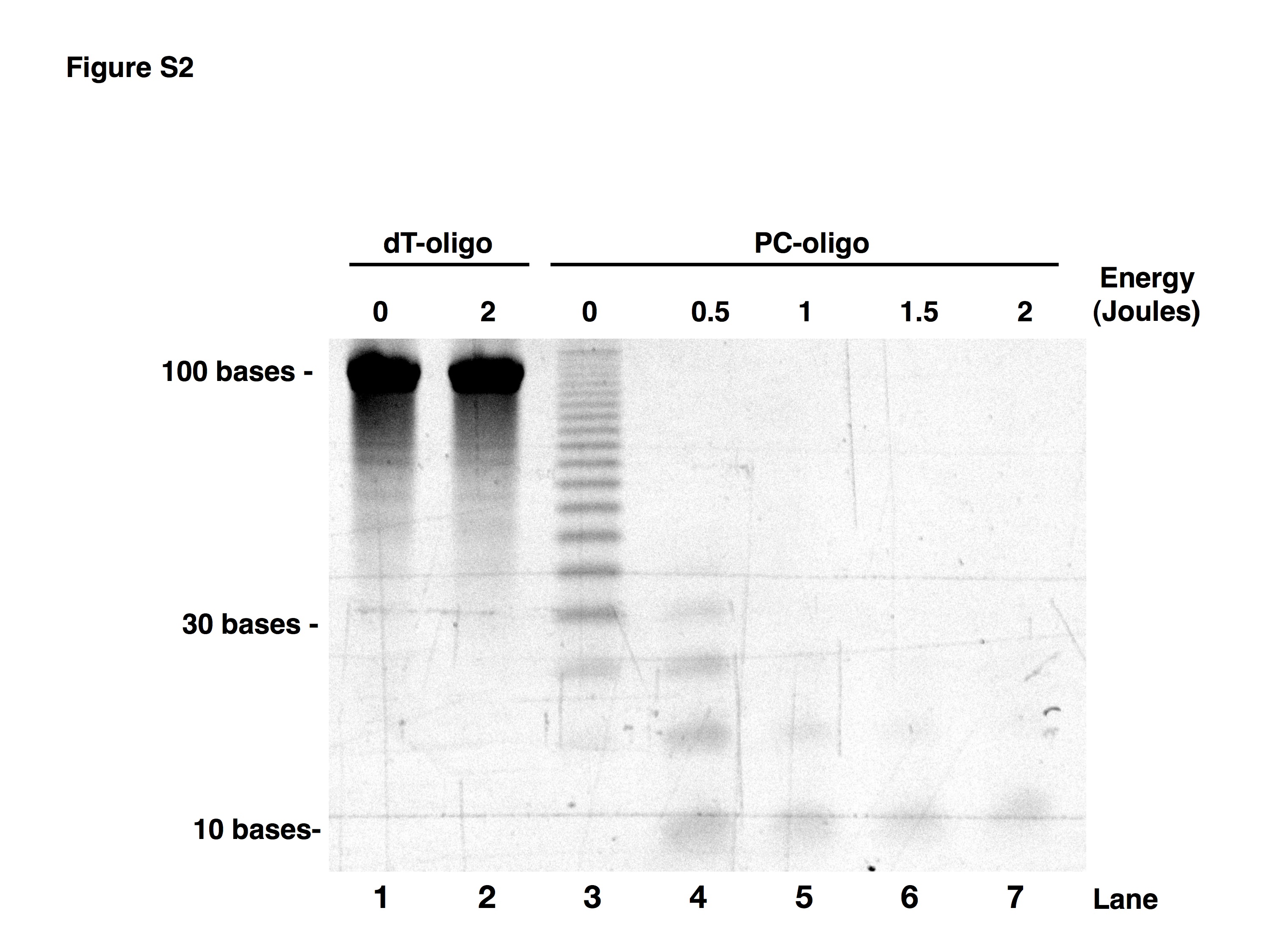


**Figure S2.** Acrylamide gel showing the effect of varying intensities of light treatment on pure oligonucleotides.


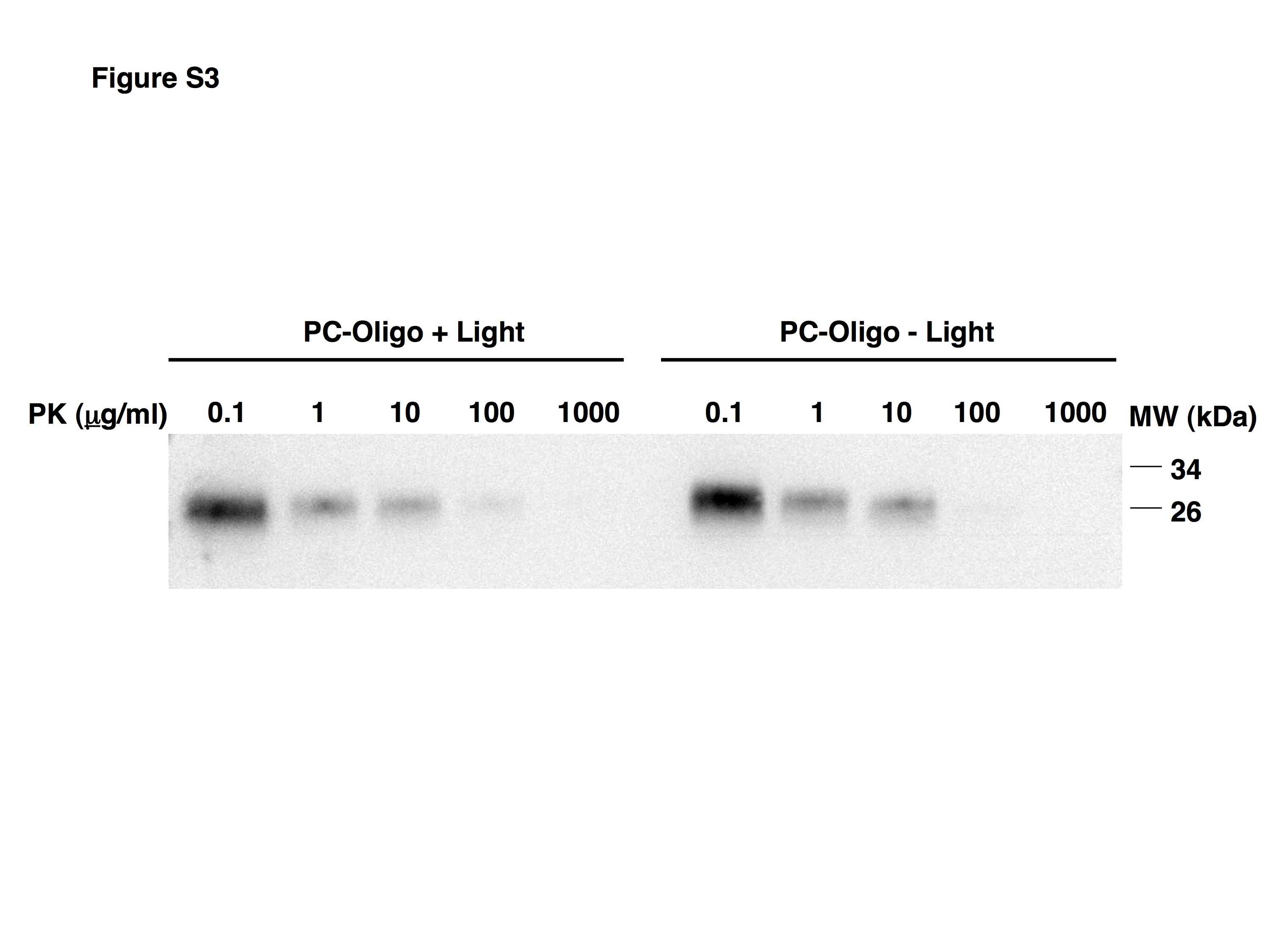


**Figure S3.** Western blot showing light-and dark-treated samples of PrP^Sc^ molecules generated with PC-oligo. Samples were treated with increasing amounts of Proteinase K as indicated for 45 min at 37°C. After digestion, samples were mixed with an equal volume of 2X SDS loading buffer and boiled for 10 min.


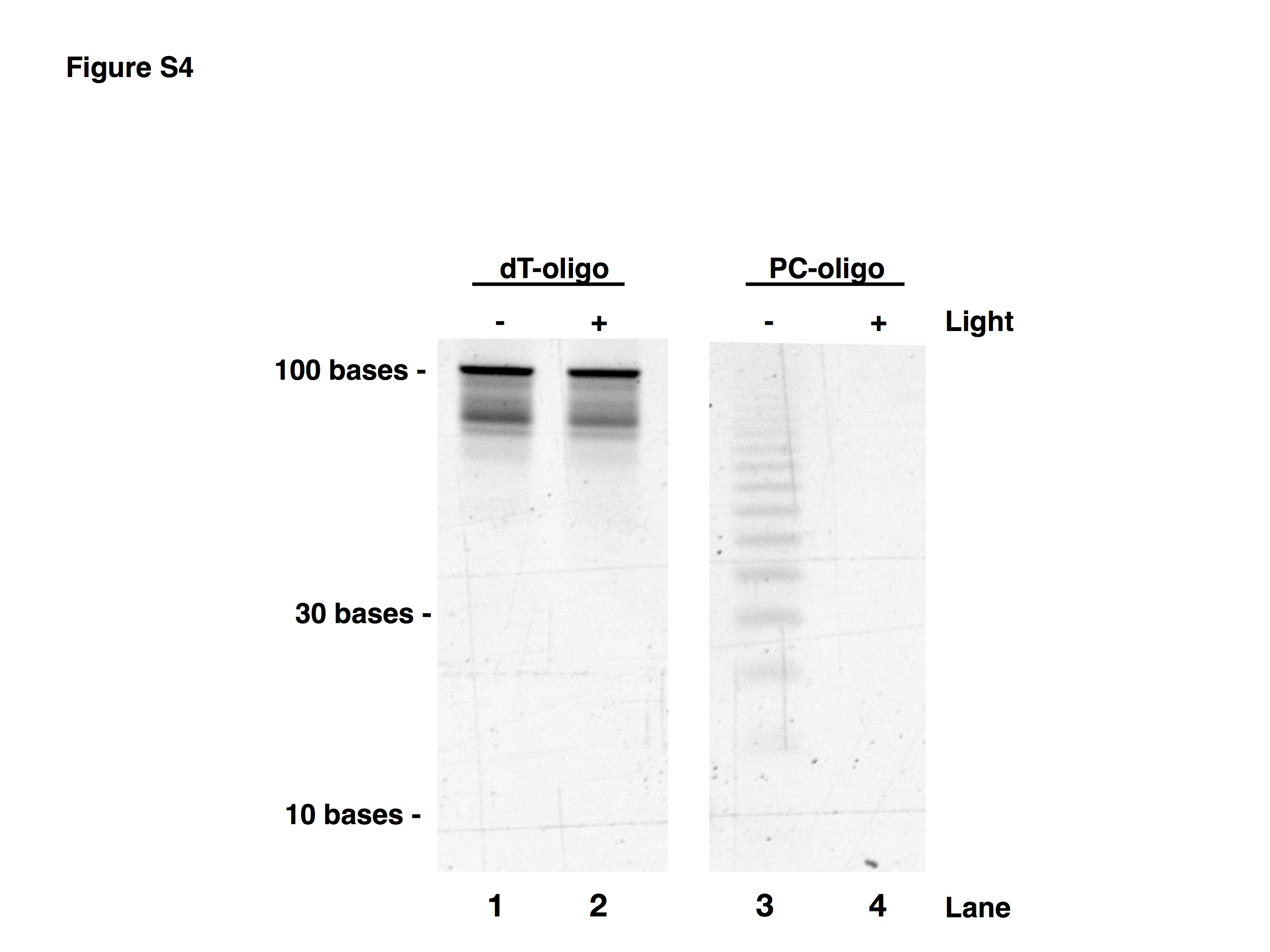


**Figure S4.** Acrylamide gel showing dT or PC-oligo recovered from aliquots of PrP^Sc^ inocula, treated with or without light as indicated.


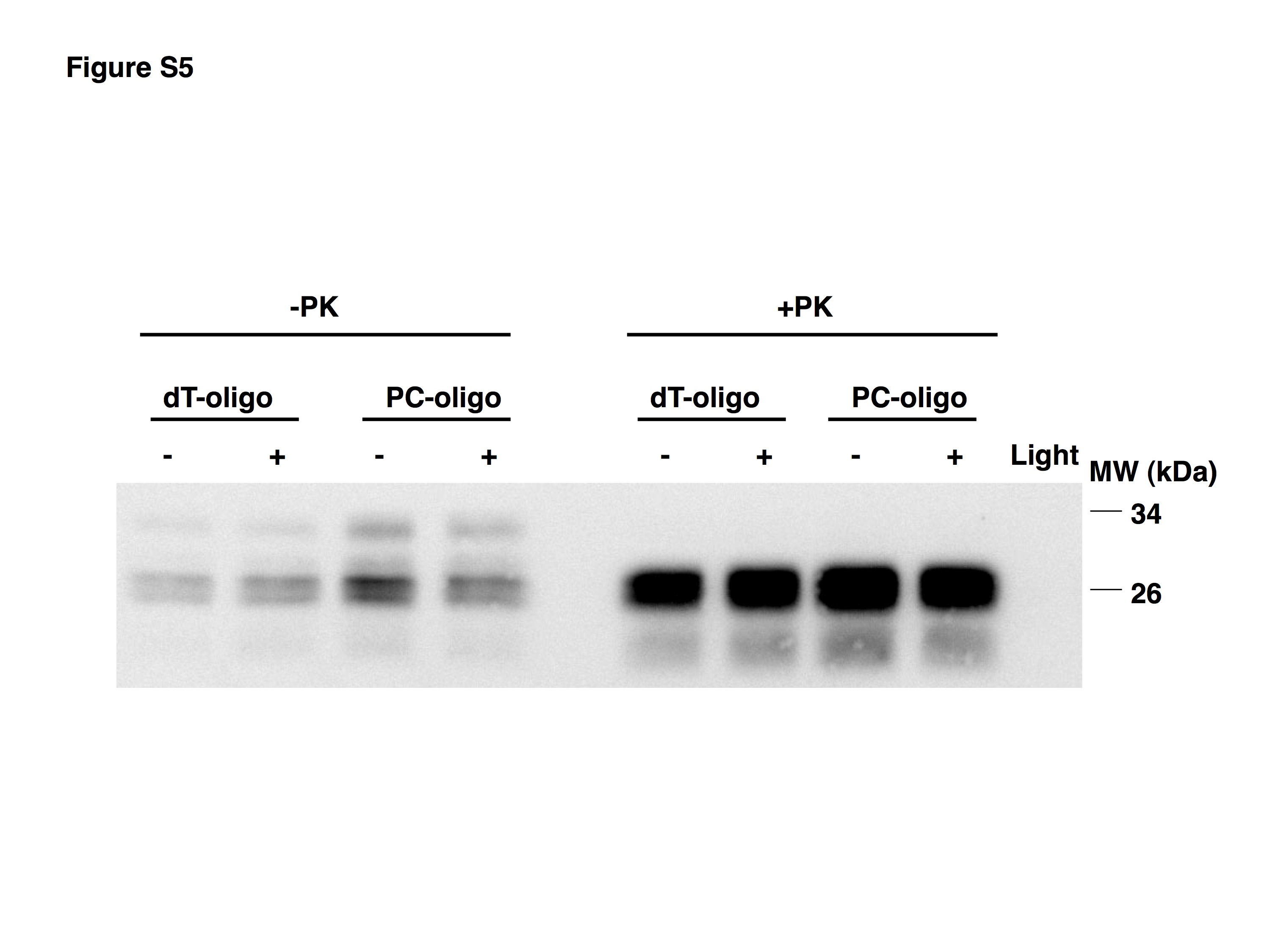


**Figure S5.** Western blot showing brain homogenates from inoculated animals. Lanes 1-4 show non-protease treated samples (-PK), 25ul loaded. Lanes 5-6 show samples subjected to limited proteolysis with proteinase K (+PK), 50ul loaded.


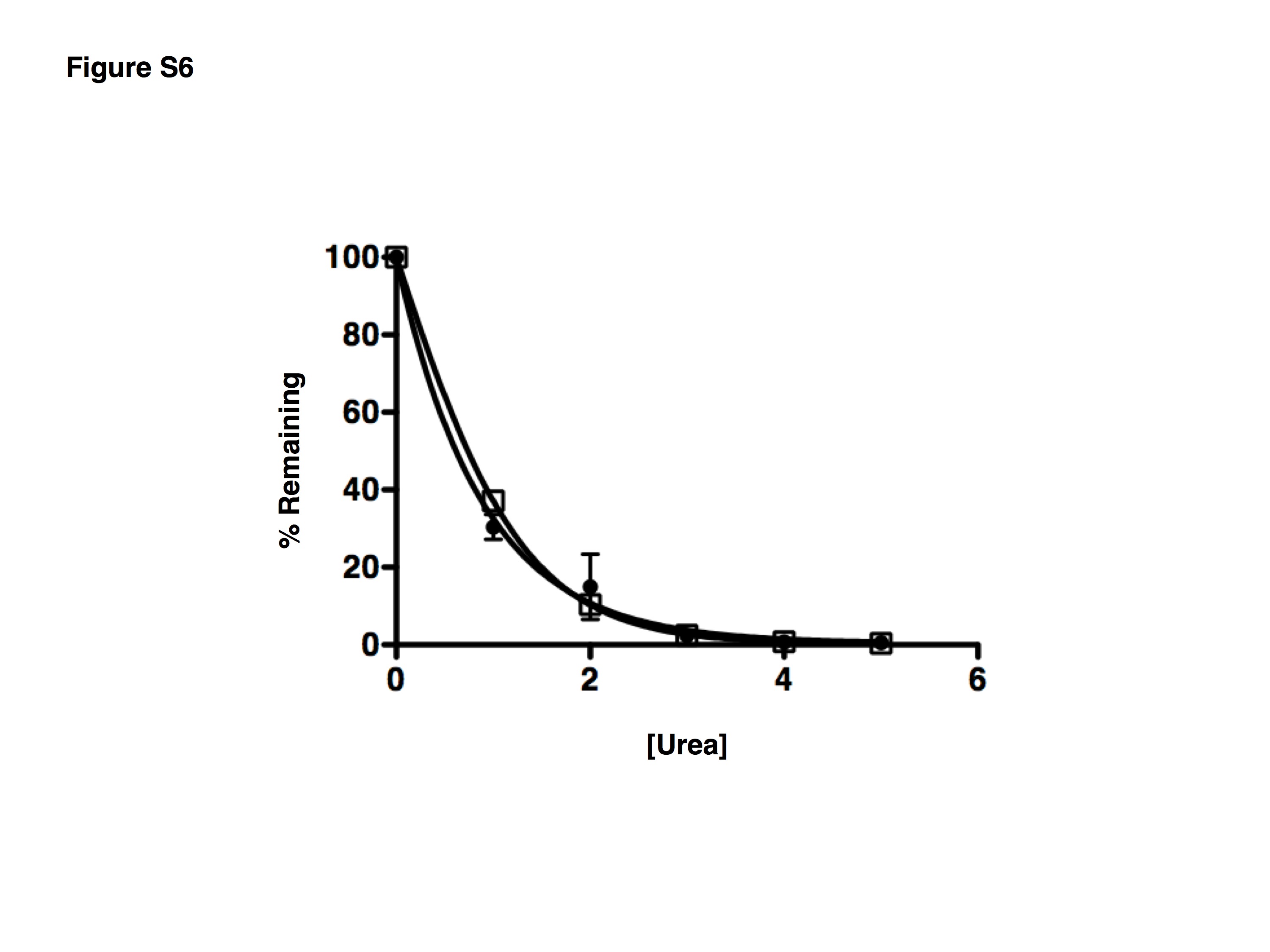


**Figure S6**. Results from urea denaturation assay using brain homogenate from animals inoculated with: (-⏺-) PrP^Sc^ molecules formed with PC-oligo and subsequently mock-treated in the dark, or (-⬜-) PrP^Sc^ molecules formed with PC-oligo and subsequently treated with UV-light. Denaturation assay was performed as previously described [1]. Western blot signals were normalized and quantified using the program Science Lab Image Guage v4.22 (FujiFilm, Tokyo, Japan). Quantified PrP^Sc^ signals were normalized to the value obtained in the absence of urea; plotted values represent the mean of 6 experiments + SEM. Signals were best fitted using a sigmoidal dose response equation with a variable slope, using the program Prism 5.0c (GraphPad Software, La Jolla, CA).

1. Piro JR, Harris BT, Nishina K, Soto C, Morales R, et al. (2009) Prion protein glycosylation is not required for strain-specific neurotropism. J Virol 83: 5321-5328.
